# Physical Activity Maintenance in the Transition to Adolescence: A Longitudinal Study of the Roles of Sport and Lifestyle Activities in British Youth

**DOI:** 10.1371/journal.pone.0089028

**Published:** 2014-02-12

**Authors:** Hannah L. Brooke, Kirsten Corder, Simon J. Griffin, Esther M. F. van Sluijs

**Affiliations:** 1 UKCRC Centre for Diet and Activity Research (CEDAR), Medical Research Council Epidemiology Unit, University of Cambridge School of Clinical Medicine, Cambridge, United Kingdom; 2 Medical Research Council Epidemiology Unit, University of Cambridge School of Clinical Medicine, Cambridge, United Kingdom; Instituto de Investigación Hospital 12 de Octubre, Spain

## Abstract

**Background:**

Promoting physical activity in youth is important for health, but existing physical activity interventions have had limited success. We aimed to inform intervention design by i) describing drop-out, continuation and uptake of specific activities over the transition to adolescence; and ii) examining Variety (number of different activities/week) and Frequency (number of activity session/week) of activity participation and their associations with changes in objectively measured physical activity from childhood to adolescence.

**Methods:**

At age 10.2±0.3 and 14.2±0.3 years, 319 children in the SPEEDY study (46% boys) wore GT1M Actigraph accelerometers for 7 days and provided self-reported participation (never, once, 2 to 3 times or four or more times, over the last 7 days) in 23 leisure-time activities. Associations of change in moderate-to-vigorous intensity PA (MVPA) (≥2000 counts/minute) and change in total physical activity (TPA) (average accelerometer counts/minute) with exposure variables Z-score transformed (change in) Variety and Frequency were examined using multilevel linear regression, clustered by school, in simple and adjusted models.

**Results:**

The number of children ever reporting a specific activity ranged from 30 (‘Hockey’) to 279 (‘Running or jogging’). Some activities were susceptible to drop-out (e.g. ‘Skipping’) but others were commonly continued or taken up (e.g. ‘Household chores’). Overall, Variety and Frequency declined (mean±SD ΔVariety −3.1±4.4 activities/week; ΔFrequency −7.2±12.0 session/week). ΔMVPA and ΔTPA were not associated with Variety or Frequency at baseline, nor with ΔVariety or ΔFrequency (p>0.29 in all models).

**Conclusions:**

Popularity of specific activities as well as drop-out, continuation and uptake should be considered in future intervention development. Activities that are commonly continued or taken up may be more valuable to encourage in interventions than those with low participation or high drop-out. We did not find evidence to support the idea that Variety and Frequency may be key elements to include in future interventions.

## Introduction

Physical activity is important for health in young people [1], however, a striking decline in physical activity, estimated to be about 7% each year, begins in childhood and continues throughout adolescence in Boys and Girls [2]–[4]. Encouraging children to maintain their activity level into their teenage years is an urgent public health challenge, particularly as interventions to date have had limited success [5].

Identifying correlates and determinants of behaviour can inform the development of evidence-based interventions [6]. A range of biological, demographic, psychological and behavioural correlates and determinants of physical activity have been studied previously in young people. Factors such as social support, self-efficacy and perceived behavioural control have been identified as potential intervention targets [7]. However, physical activity is a complex behaviour and looking beyond traditional correlates and determinants extends the current understanding and further informs intervention design.

Intervention development may be aided by understanding changes in the characteristics of physical activity behaviour from childhood to adolescence, for example the changing participation in specific activities. Activities that children often drop out of may be best avoided in interventions. Activities which children commonly continue or take-up could be salient to promote. Longitudinal studies report declining participation in specific activities, with more prominent declines in some activities than others, depending on the activity and cultural context [8]–[10]. Drop-out, continuation and uptake of specific activities have not previously been described through the transition from childhood to adolescence in British youth.

The dimensions of physical activity themselves, such as the number of different types of activity participated in each week (Variety), and how often activities are participated in (Frequency), could be considered correlates and determinants of physical activity. Children who participate in greater Variety or Frequency of activities may be more protected from the decline in physical activity into adolescence than others. Variety or Frequency could then be targeted in interventions. Variety has a greater annual decline than other dimensions of physical activity [4] suggesting importance; however, further research is required to confirm this observation. Moreover, whether (change in) Variety is associated with changes in overall physical activity is unknown. A lower Variety may indicate *specialisation*, i.e. fewer activities participated in with greater frequency, rather than lower overall activity. It is therefore important to examine Variety and Frequency simultaneously and attempt to disentangle the relationship between these two dimensions and overall physical activity level. Cross-sectional analyses indicate that Variety and Frequency are associated with objectively measured physical activity, although not independently [11]. However, longitudinal studies are required to examine whether Variety and Frequency have a role in physical activity maintenance.

Existing longitudinal studies indicate that the decline in physical activity in adolescence is due to participation in a smaller Variety of activities rather than less time in each activity [12]. Moreover, Variety at age 15 years and physical activity at age 23 years are positively correlated [9] and Variety in early adolescence predicts change in physical activity [13]. This research supports the idea that Variety may be important for physical activity maintenance in young people and is a construct that merits further research. However, unlike the current study these observations are limited by the self-reported nature of the physical activity measure and do not take Frequency into account. Objective measures of physical activity provide more accurate effect size estimates [14] and may reduce correlated error. One cross-sectional study in children that used objectively measured physical activity as an outcome did not find an association with Variety [15]. Using objective measures of physical activity to clarify the conflicting findings of previous studies and assess the role of Variety and/or Frequency in the maintenance of physical activity in young people is therefore important.

We aimed to explore drop-out, continuation and uptake of specific activities in youth. These characteristics of physical activity have not been described in a British population and could aid context specific intervention development by highlighting salient activities to promote. We also aimed to assess changes in Variety and Frequency of activities out of school and their associations with change in objectively measured physical activity. Our broad hypothesis was that children with higher baseline, or smaller decreases in Variety and/or Frequency of out-of-school activities would experience less reduction in objectively measured physical activity than children with lower baseline, or larger decreases in Variety and/or Frequency. This research will expand current knowledge because in addition to a longitudinal design we will use an objective measure of physical activity as an outcome. Moreover, models will include both Variety and Frequency as exposures which will account for the possibility of *specialisation*.

## Methods

### Ethics Statement

Full ethical approval was received from the University of East Anglia local research ethics committee.

### Study Outline

The Sport, Physical activity and Eating behaviour: Environmental Determinants in Young People (SPEEDY) study is a population-based longitudinal cohort study of children from primary schools in Norfolk, Eastern England. Baseline recruitment and methods of the study have been described previously [16]. In short, information about the study was sent to 157 out of 227 (69%) primary schools in Norfolk with more than 12 pupils enrolled in Year-5 (children aged 9 to 10 years). Schools were purposively sampled to achieve heterogeneity in urban/rural location; within locational strata, the schools were randomly selected to be invited. Ninety-two schools participated. Overall, 2064 pupils (57% of eligible sample) returned parental consent forms and participated in baseline measurement from April-July 2007. Four years later participants with an active postal address and who had not withdrawn from the SPEEDY study (n = 1964) were contacted via their home address. Parents and children provided written informed consent and participated in follow-up data collection between April-July 2011. Information about the weather during data collection was derived from two of the UK Meteorological Office's MIDAS Land Surface Observation Stations in Norfolk [17]. At baseline mean(SD) rainfall = 1.80(3.37)mm 7am–9pm, maximum daily temperature = 17.46(3.23)deg C, day length = 16:02:36(00:50:30)hr:min:sec. At follow up mean(SD) rainfall = 1.03(2.57)mm 7am–9pm, maximum daily temperature = 19.07(3.17)deg C, day length = 16:08:15(00:53:57)hr:min:sec. The longitudinal sample in the current study comprised of 415 children with data at both baseline and follow-up.

At both time points all children were visited in school. Trained research assistants followed standard operating procedures to conduct anthropometric measurements, supervise the completion of questionnaires and fit each child with an accelerometer which was to be returned to school eight days after the measurement day. At baseline children also took a questionnaire home for their parent or guardian; this was returned to the schools.

### Youth Physical Activity Questionnaire (YPAQ)

**Figure 1:**
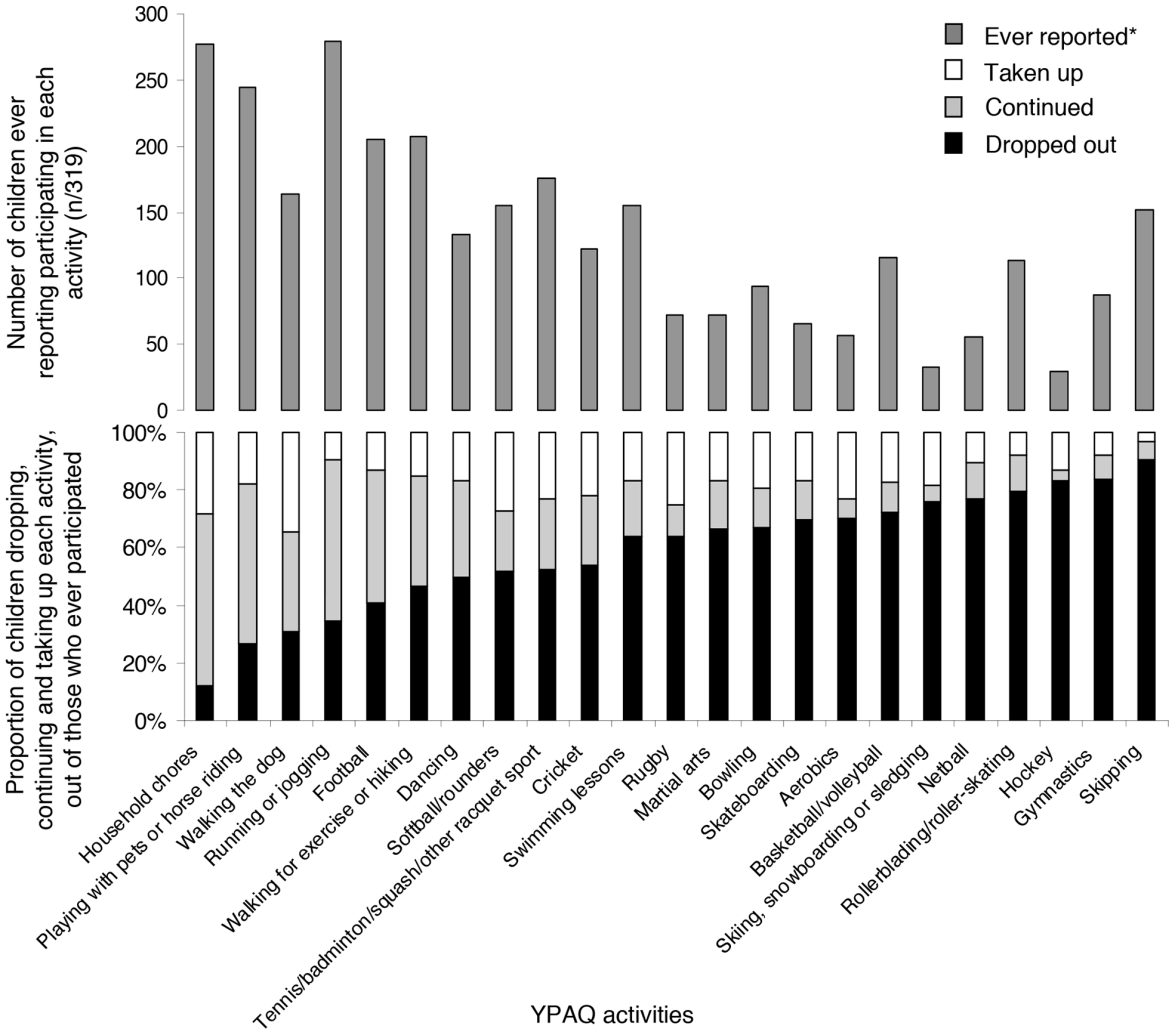
Changes in children's participation in specific activities reported in the Youth Physical Activity Questionnaire. Number of children who ever reported participating in each Youth Physical Activity Questionnaire (YPAQ) activity (top panel) and the proportion of children who dropped out of, continued or took up each YPAQ activity between baseline and follow-up measurements, (percentage of those who ever reported participating in it) (bottom panel). *reported to be participated in during the 7 days before the questionnaire was completed at either time point.

At baseline and at follow-up children self-reported their involvement in common moderate-to-vigorous intensity activities outside school over the preceding seven days using the YPAQ. There were four response categories for each activity (never, once, 2 to 3 times and four or more times); these were recoded as 0, 1, 2.5 and 4.5 sessions/week for analysis. The YPAQ was adapted from the Children's Leisure Activities Study Survey (CLASS) [18] for a British population and is able to rank individual's overall physical activity [19]. In total, 23 activities were available at both time points (**Figure 1**). The YPAQ gave an opportunity for children to report ‘other’ activities they participated in. Activities reported as ‘other’ but that were covered by an activity listed in the YPAQ were recoded as appropriate. Only 7 children reported doing the same ‘other’ activity at baseline and follow-up. Due to heterogeneity in types of ‘other’ activities, they were not included in analyses. Activity drop-out (activity reported at baseline but not follow-up), continuation (activity reported at baseline and follow-up), and uptake (activity not reported at baseline but reported at follow-up), were calculated for each child for each specific activity and presented as a proportion of children ever reporting participation.

Using data from the YPAQ, Variety (the sum of activities reported to be participated in) and Frequency (summed sessions per week of all activities reported) were derived. To allow the comparison of β-coefficients across exposures, z-scores transformations of Variety and Frequency 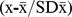 were applied.

Children were excluded from analyses if data for more than one third of activities (i.e. 7/23) were missing (baseline, n = 10/415; follow-up, n = 5/415). For all other children we assumed that no response equated to never participating in that activity.

### Accelerometry

At both time points ActiGraph accelerometers (GT1M, Actigraph LCC, Pensacola, US) [20] were used to assess physical activity in normal daily life, data were stored in 5-second epochs [21]–[23]. In children and adolescents uniaxial accelerometers explain a moderate proportion (33 to 69%) of the variance in physical activity in normal daily life derived from doubly labelled water [24]. Children were asked to wear the accelerometer on an elastic waistband on the right hip for seven days and to remove it only while sleeping, bathing, showering and swimming [20]. Custom software (MAHUffe, http://www.mrc-epid.cam.ac.uk) was used for data processing. Our processes were similar to other studies in young people [25]–[27] and identical at both time points. Data from the day the accelerometer was fitted, periods with ten or more minutes of sustained zero counts (‘non wear time’) and counts before 6am and after 11pm each day were removed from all files. Children who did not obtain at least 500 minutes of valid accelerometer data per day on at least three days at each time point were excluded from analyses (baseline, n = 26/415; follow-up, n = 57/415). Three days of physical activity monitoring with uniaxial accelerometers has previously given a reliability coefficient of 0.70 in children of a similar age [27], [28].

We defined MVPA as time (minutes) spent at ≥2,000 counts per minute (cpm) each day. This is approximately equivalent to a walking pace of 4 km/h in children [29] and has been used in previous studies of children [30]. Total physical activity level (TPA) reflects average accelerometer cpm; it was calculated as total counts divided by total valid monitoring time. MVPA and TPA are presented as a change in daily mean value (follow-up minus baseline).

### Measurement of covariates

Date of birth was self-reported and age was calculated from the measurement date. Height was measured to the nearest 0.1 cm (Leicester height measure, Chasmors Ltd., Leicester, UK). A non-segmental bio-impedance scale (Tanita, type TBF-300A, Tokyo, Japan) was used to measure weight to the nearest 0.1 kg with children dressed in light clothing. Body mass index (BMI) (kg/m^2^) was calculated and age-standardised BMI was derived. Obesity status was dichotomised (non-overweight vs. overweight/obese) based on sex- and age-dependent cut points [31].

At baseline parent/guardians self-reported their highest educational qualification; this was used as a proxy measure of socioeconomic status. A categorical variable (parent/guardian education level) with 3 groups was created, ‘GCSE or lower’ (i.e. no educational qualification, school leaving certificate, General Certificate of Secondary Education; GCSE, or equivalent), ‘A-level or lower vocational training’ and ‘University or higher vocational training’. Children were excluded from analyses if they had missing data at baseline for age (n = 0/415), sex (n = 0/415), BMI (n = 2/415), or parent/guardian education level (n = 14/415).

### Statistical analyses

To account for the sampling framework ‘school’ was a multilevel cluster variable in all analyses. Complete data for exposures, outcomes and covariates were available for 319 out of 415 children. Differences in baseline characteristics between children included in the current analyses (n = 319) and those with baseline data available (n = 1745) and sex differences in baseline characteristics were tested using multilevel linear or multinomial logistic regression according to whether the dependent variable was continuous or categorical. Variables and their residuals were judged to be normally distributed so were not transformed. Change in Variety, Frequency, MVPA and TPA (follow-up minus baseline), and sex difference in change (change x sex interaction), were also tested using multilevel linear regression. Multilevel linear regression was used to assess whether change in physical activity (MVPA and TPA in separate models) was associated with Variety and/or Frequency at baseline or change in Variety and/or Frequency between baseline and follow-up. Variety and Frequency were included in separate models and together in mutually adjusted models to test the independence of any associations. In addition, analyses were run in simple models and in models adjusted for potential confounders measured at baseline: age, sex, parent/guardian education level, age-standardised BMI and TPA.

Three sensitivity analyses were conducted for associations of Variety and/or Frequency with objectively measured physical activity. Firstly, using objectively measured physical activity for the out-of-school time period only; secondly, excluding all children with any missing YPAQ data at either time point, and thirdly, creating categorical variables for Variety and Frequency based on tertiles and using these instead of continuous variables.

## Results

Children included in the current analysis (n = 319) were of comparable age, height, weight and BMI, and had similar parent/guardian education levels and activity levels (p<0.05) at baseline to those who were not included in the current analysis (n = 1745). Boys and girls in the current analysis sample had similar baseline personal, anthropometric and demographic characteristics (**Table 1**).

**Table 1:** Baseline personal, anthropometric and demographic characteristics of children from the SPEEDY study whose data were used in these analyses.

		Boys (n = 146)	Girls (n = 173)	Total (n = 319)	P-value (sex differences)
**Age (years)**	Baseline	10.2±0.3	10.3±0.3	10.2±0.3	0.616
	Follow-up	14.3±0.3	14.3±0.3	14.3±0.3	0.538
**Height (cm)**	Baseline	140.9±6.1	140.3±6.7	140.6±6.4	0.398
	Follow-up	166.9±7.5	162.5±6.1	164.5±7.1	<0.001
**Weight (kg)**	Baseline	35.9±7.6	36.1±8.8	36.0±8.2	0.806
	Follow-up*	56.5±12.7	56.5±11.4	56.5±12.0	0.987
**BMI (kg/m^2^)**	Baseline	17.8±2.8	18.1±3.2	18.0±3.0	0.501
	Follow-up*	20.1±3.6	21.3±3.9	20.8±3.8	0.004
**Parent/guardian education level**					
GCSE or lower	Baseline	37 (25)	58 (34)	95 (30)	Ref
A-level or lower vocational	Baseline	74 (51)	80 (46)	154 (48)	0.090
University or higher vocational	Baseline	35 (24)	35 (20)	70 (22)	0.126
**Weight status**					
Non-overweight	Baseline	121 (83)	133 (77)	254 (80)	Ref
	Follow-up*	120 (83)	134 (78)	254 (80)	Ref
Overweight/obese	Baseline	25 (17)	40 (23)	65 (20)	0.200
	Follow-up*	25 (17)	38 (22)	63 (20)	0.305

Data are presented as mean ± standard deviation for continuous variables and n (%) for categorical variables.

Ref is the reference category.

Differences between Girls and Boys (p-value presented) were determined using multilevel linear or logistic regression depending on whether the dependent variable was continuous or categorical.

SPEEDY, Sports, Physical activity and Eating behaviour: Environmental Determinants in Young people; BMI, body mass index; GCSE, General certificate of secondary education.

*At follow-up one boy and one girl were missing data for weight therefore weight, BMI and weight status at follow-up are out of 317 children (145 boys, 172 girls)

Activities varied widely in total participation; out of 319 children, those ever reporting participation in a specific activity ranged from 30 (‘Hockey’) to 279 (‘Running or jogging’). Activity specific variation was also observed in drop-out, continuation and uptake, between age 10 and age 14 years (**Figure 1**). While some activities were susceptible to drop-out (e.g. ‘Skipping’) others were commonly continued or taken up (e.g. ‘Household chores’). On average (median(IQR)) 60(43–75)% of activities reported at age 10 were dropped by age 14, this equates to 4(2–7) activities, while only 3(2–4) activities were continued. Of the activities reported at age 14, 33(17–50)% were new since age 10, this represents 1(1–3) activities.

**Table 2** shows descriptive characteristics of Variety, Frequency and objectively-measured physical activity. There were no sex differences in Variety or Frequency at either time point, but significant declines in both were observed in boys and girls. Boys had higher MVPA and TPA than girls at both time points. MVPA and TPA declined significantly between the two measurements; the decline in MVPA was stronger in boys than girls.

**Table 2:** Variety and Frequency of YPAQ activities and physical activity characteristics of boys and girls measured at baseline and follow-up.

	Boys (n = 146)	Girls (n = 173)	Total (n = 319)	P-value (sex differences)	P-value (wave differences)
**Variety (number of activities per week)**					
Baseline	7.4±4.2	8.2±4.2	7.9±4.2	0.091	-
Follow-up	4.8±2.5	4.7±2.3	4.7±2.4	0.939	-
Change	−2.7±4.2	−3.5±4.5	−3.1±4.4	0.096*	<0.001
**Frequency (number of activity sessions per week)**					
Baseline	16.8±11.8	17.9±11.8	17.4±11.8	0.391	-
Follow-up	10.3±6.2	10.1±5.8	10.2±6.0	0.788	-
Change	−6.5±11.6	−7.8±12.4	−7.2±12.0	0.327*	<0.001
**TPA (cpm)**					
Baseline	724.5±260.8	638.7±214.0	678.0±240.1	0.000	-
Follow-up	531.4±169.5	463.2±201.1	494.4±190.1	0.003	-
Change	−193.1±261.5	−175.5±283.4	−183.6±273.3	0.564*	<0.001
**MVPA (mins/day)**					
Baseline	83.8±26.5	67.9±20.0	75.1±24.5	0.000	-
Follow-up	67.1±24.7	59.5±23.3	63.0±24.2	0.011	-
Change	−16.7±29.0	−8.3±25.9	−12.2±27.7	0.006*	<0.001

Data are presented as mean ± standard deviation.

Differences between girls and boys (p-value for sex differences) and differences between baseline and follow-up measurements (p-value for wave differences) were determined using multilevel linear regression.

*P-value for wave x sex interaction.

YPAQ, youth physical activity questionnaire; TPA, total physical activity; cpm, counts per minute; MVPA, moderate-to-vigorous intensity physical activity.

Although Variety and Frequency were strongly correlated (r = 0.87, 0.83 and 0.85 for baseline, follow-up and change respectively) multi co-linearity was not judged to be influential for any regression analyses (Variance Inflation Factors ranged from 1.68 to 4.08). There were no associations of change in MVPA or change in TPA with baseline Variety or baseline Frequency, or with change in Variety or change in Frequency in simple or adjusted models (**Table 3**) (all models p>0.29).

**Table 3:** Associations of change in objectively measured MVPA and change in TPA between baseline and follow-up with self-reported Variety and Frequency measured at baseline and change in self-reported Variety and Frequency between baseline and follow-up.

		Variety and Frequency in separate models				Variety and Frequency in the same (mutually adjusted) model			
		β Coef.	95% CI		P-value	β Coef.	95% CI		P-value
**Change MVPA**	Baseline Variety	−0.35	−3.62	2.92	0.831	−2.01	−7.34	3.32	0.456
	Baseline Frequency	0.16	−2.92	3.25	0.917	1.90	−3.04	6.83	0.447
**Change TPA**	Baseline Variety	7.99	−16.85	32.84	0.524	−5.42	−39.19	28.35	0.750
	Baseline Frequency	10.68	−18.98	40.34	0.476	15.36	−31.90	62.63	0.519
**Change MVPA**	Change Variety	0.61	−1.73	2.96	0.605	−0.03	−3.72	3.65	0.985
	Change Frequency	0.75	−1.91	3.42	0.575	0.78	−3.55	5.12	0.720
**Change TPA**	Change Variety	2.35	−13.20	17.89	0.765	13.95	−18.45	46.35	0.394
	Change Frequency	−2.37	−27.00	22.26	0.848	−14.06	−61.00	32.88	0.553

Presented models were adjusted for school, age, sex, parent/guardian education level, age standardised body mass index, and baseline TPA.

Variety (number of activities per week) and Frequency (number of activity sessions per week) variables were transformed to z-scores for analyses; therefore effect sizes are directly comparable.

Associations were tested using multilevel linear regression.

P–value for significance of the association of baseline Variety, baseline Frequency, change in Variety or change in Frequency with change MVPA or change TPA.

MVPA, moderate-to-vigorous intensity physical activity; TPA, total physical activity; β Coef., β Coefficient; 95% CI, 95% confidence interval.

Sensitivity analyses did not show different results from the main analyses if objectively measured physical activity excluding school-time was used, or if all children with any missing YPAQ data were excluded. The results when considering tertiles of Variety and Frequency were largely unchanged except for the association between change in TPA and change in Frequency, adjusted for change in Variety. This model indicated that children in the highest tertile for change in z-score Frequency had significantly less of a change in objectively measured TPA compared with those in the lowest tertile.

## Discussion

This study is the first, to our knowledge, to examine changes in specific activity participation from childhood to adolescence in British youth. Our results improve the understanding of changes in the characteristics of physical activity behaviour by using a longitudinal design, an objective measure of physical activity as an outcome and including Variety and Frequency in the same models. We highlight salient activities for physical activity promotion, but found no evidence that Variety and Frequency of YPAQ activities, participated in by young people out-of-school, could be key for physical activity maintenance.

Activity popularity is often culture specific, for example Basketball is popular in North America [12], [32], but only reported by 36% of children in the current sample. However, ‘Running or jogging’ was the most commonly reported activity and is popular in other cultural contexts [10], [12], [15], [32], [33]. ‘Household chores’ and ‘Playing with pets or horse riding’ were also commonly reported. Lifestyle activities may make an important contribution to physical activity and thus could represent a feasible route to enable activity maintenance throughout adolescence and continued into adulthood. Identifying popular activities amongst young people highlights potential activities to promote in interventions. However, the culture and context of the intervention population should be taken into account. This study is therefore particularly important for the development of physical activity interventions in British youth.

Alongside the variation in overall popularity there was variation in activity drop-out, continuation and uptake over time. Broadly, our results agree with the findings of others [12]; with high drop-out and sustained non-participation but low uptake and continuation of activities. Activities varied widely in the degree to which participation changed over time. This observation is supported by some previous cross-sectional [15] and longitudinal research [13]. For example, between ages 11 and 15 years the proportion of boys who reported participating in ‘Volleyball’ halved, but the pattern was reversed for ‘Indoor soccer’ [13]. While the specific activities and direction of change differed between the previous studies and the current study (perhaps because of differing age groups and contexts), changes in participation were evidently activity-specific. Conversely, other studies indicate that participation declines uniformly across all activities. However, these studies have been in older children than the current sample [10], [34], which may indicate there is a plateau in the changes in activity types after puberty.

Detailed examination of changes in participation in specific activities can inform intervention design, although overall participation should be kept in mind. Activities with high drop-out or low continuation and uptake could represent targets for interventions as there is greater scope to improve maintenance of participation. However, this strategy may be problematic if participation patterns are due to low social acceptability or redundancy; for example, ‘Swimming lessons’ may be unnecessary if proficiency has been achieved. Conversely, activities with high continuation, such as ‘Running and jogging’, are evidently feasible to maintain and illustrate alternative target activities for interventions. Uptake of activities such as ‘Walking the dog’ may be related to age dependant responsibilities but uptake fits within social norms; these activities perhaps also represent relevant activities to promote. Qualitative research exploring *why* drop-out, continuation and uptake vary between activities would help clarify these conflicting arguments and could further inform future intervention design.

The decline in overall Variety in this study, from 7.9±4.2 to 4.7±2.48 (mean±SD change −3.1±4.4) activities per week between ages 10 and 14 years, was similar to that previously reported in the USA in children of comparable age (mean change −3.97±2.91 activities) over four years [12]. However, others have reported a greater Variety of activities in an older cohort of children (aged 10–12 years) compared with a younger cohort (aged 5–6 years) [15]. One explanation for these differing results could be that Variety reaches a peak towards the end of childhood before declining during adolescence. Longitudinal research following younger children over a longer time than the current study would be necessary to investigate this possibility.

Converse to previous longitudinal studies [9], [12], [13] we found no evidence linking Variety of out-of-school activities to physical activity maintenance. Existing longitudinal studies indicate that the decline in physical activity in adolescence is due to participation in a smaller Variety of activities rather than less time in each activity [12]. Moreover, Variety at age 15 years and physical activity at age 23 years are positively correlated [9] and Variety in early adolescence predicts change in physical activity [13]. However, these studies used self-reported measures of physical activity and did not take into account changes in Frequency. The only other study, to our knowledge, which has examined the relationship between Variety and objectively measured physical activity also did not find an association [15]. This was a cross-sectional analysis conducted in younger children using a proxy-report of Variety rather than self-report [15]. The agreement with our results indicates that using an objective measure for the outcome could explain conflicts between previous studies. Differences in the population context and age ranges studied provide alternative explanations for discrepancies.

Although we found no evidence that the Variety and Frequency of YPAQ activities participated in by children outside of school are key for physical activity maintenance, Variety and Frequency remain important characteristics of physical activity as they are vital for maintaining a balance between cardio-metabolic health and bone health in young people [35]. It is not yet feasible to objectively measure types of activity on a large scale, but when it is, repeating this study with objectively measured exposures may be valuable. Mismatches in the accuracy of exposure and outcome measurement could obscure potential associations. As YPAQ activities were associated with objectively measured MVPA cross-sectionally, we do not think that a low correspondence between the YPAQ and actual or typical behaviours that children engage in is a central reason for the lack of association. However, future research should also consider Variety and Frequency of activities which were not captured by the YPAQ. Formative research could be used to develop population-appropriate questionnaires. In addition, using direct observation, ecological monitoring or qualitative methods could extend the results of this study by capturing non-specific physical activity behaviours such as active play [36] which may be difficult for children to recall in a questionnaire.

The first two sensitivity analyses showed that the results were not influenced by modifying the outcome or the exposure, which adds confidence to the main findings. Using a categorical rather than a continuous variable for Variety and Frequency also largely supported the main analysis. The one model that did change suggests that children with a lower decline in Frequency also had a lower decline in physical activity which reflects our original hypothesis.

Our study had strengths as it was a longitudinal, population-based sample with objectively measured physical activity outcome, however, several limitations should be taken into account. A large proportion of children were lost to follow-up; however, baseline characteristics suggest that the current sample were largely representative of the original cohort. Despite this, the results should be generalised to this and other populations with caution, particularly because the population of Norfolk is not representative of the rest of the UK. Accelerometers cannot be worn for water-based activities and do not accurately reflect activities with limited vertical hip movement (e.g. cycling), therefore they underestimate physical activity in children who regularly swim or cycle. Self-reported and objective physical activity were assessed in consecutive weeks which possibly weakened any relationship that may have been between the two measures. In addition, the YPAQ only captured non-school activities, whereas objectively measured physical activity was determined over the whole day. This approach was chosen as we are primarily concerned with developing interventions that facilitate children to increase their activity level overall so it is important to know about activity over the whole day. Of 28 activities in the YPAQ at baseline only 23 were also in the YPAQ at follow-up. We are unable to comment on the changes in participation in the 5 unavailable activities and other activities which were not captured by the YPAQ; nor able to comment on the associations of physical activity with Variety and Frequency of such activities. An additional strength of our study is using identical methods at both time-points. However, cognitive development between measurement waves may alter interpretation, perspectives or memory of events. This is a limitation of all longitudinal studies in young people and could influence the descriptive results of activity drop-out, continuation and uptake as well as Variety and Frequency measures. The low participation observed for ‘Netball’, ‘Hockey’ and ‘Skiing, snowboarding or sledging’ could be because of seasonality of activities. Our data were collected in spring/summer whereas these activities are traditionally participated in during autumn/winter in the UK and our results therefore may not be generalisable to other times of the year. This further illustrates the importance of the context in which an intervention is designed to operate in. It emphasises the need for flexible and adaptive interventions that can be shaped to the situation.

In conclusion, popularity of specific activities as well as drop-out, continuation and uptake should be considered in intervention development, although ensuring interventions are appropriate for the context and culture in which they will be implemented is important. Activities with low participation or that are often dropped may not reflect wise choices of activities to emphasise in interventions, but it may be prudent to encourage activities that are commonly continued or taken up. Contrary to previous studies we did not find evidence that Variety and Frequency of out-of-school activities play a role in activity maintenance in young people. The Variety and Frequency of YPAQ activities engaged in by children outside of school therefore do not appear to be key elements to include in interventions and public health guidelines to encourage the maintenance of physical activity from childhood to adolescence. However, they should not be dismissed as they perform other essential functions related to physical health.
